# Neural Network Command Filtered Control of Fractional-Order Chaotic Systems

**DOI:** 10.1155/2021/8962251

**Published:** 2021-10-21

**Authors:** Hua Zhang

**Affiliations:** Zhengzhou Preschool Education College, Zhengzhou 450099, China

## Abstract

An adaptive neural network (NN) backstepping control method based on command filtering is proposed for a class of fractional-order chaotic systems (FOCSs) in this paper. In order to solve the problem of the item explosion in the classical backstepping method, a command filter method is adopted and the error compensation mechanism is introduced to overcome the shortcomings of the dynamic surface method. Moreover, an adaptive neural network method for unknown FOCSs is proposed. Compared with the existing control methods, the advantage of the proposed control method is that the design of the compensation signals eliminates the filtering errors, which makes the control effect of the actual system improve well. Finally, two examples are given to prove the effectiveness and potential of the proposed method.

## 1. Introduction

The fractional calculus equations are increasingly used to describe problems in optical and thermal systems, material and mechanical systems, signal processing, system identification, control, robotics, and other applications. The theory of fractional calculus has also been paid more and more attention by scholars at home and abroad, and especially, the fractional differential equations abstracted from practical problems have become the research focus of many mathematicians [1–3]. Bao and Cao [4] proved that fractional-order differential equations can well simulate the dynamics of various chemical materials and special materials in practical applications. The traditional integer-order differential equation can be regarded as a special model of fractional-order differential equation. More information on fractional-order differential integration can be found in [5, 6]. In recent years, many achievements have been made in the study of fractional-order calculus equations, which are used to study the problems of stability analysis and control of fractional-order strictly feedback nonlinear systems. For example, Li et al. [7] proposed a Lyapunov direct method for fractional-order nonlinear systems. Boroujeni and Momeni [8] proposed a fractional-order state observer for fractional-order nonlinear systems. Yang and Chen [9] presented a finite-time stabilization method for fractional-order switching systems. Adaptive control methods had been used in the control of fractional-order nonlinear systems [5, 6, 10]. Huang et al. [11] proposed a fuzzy feedback control method for uncertain fractional-order chaotic systems (FOCSs). Therefore, the research on the stability and control of FOCSs is becoming more and more popular.

The backstepping control was proposed for the first time to obtain asymptotic tracking and global stability of strictly feedback nonlinear systems [12]. The backstepping technique has always been a powerful tool for the design of controllers for nonlinear dynamic systems [13, 14]. However, the classical backstepping control has two obvious disadvantages: one is the explosion of terms caused by the repeated derivation of virtual control signals [15–17], and the other is certain functions in the systems with uncertain parameters must be linear [18–20]. Li et al. [20] presented a fuzzy adaptive output feedback control method to improve the tracking effect of the systems. In addition, Shao-Cheng Tong et al. [21] proposed a backstepping method based on command filters to solve the problem of item explosion caused by repeated derivation of the virtual controller. Then, the command filtered backstepping control was extended to the adaptive case in [22]. In each step of the control design, the output of the command filter was used to approximate the differential coefficient of the virtual control signal, which eliminates the problem of item explosion. Note that the error compensation mechanism was designed to eliminate errors generated by the command filters [23]. However, the above works considered only the cases of systems with unknown parameters which are constant and systems without unknown parameters. In addition, the above research results only consider the case of integer-order systems, but the research results of the backstepping control method based on command filtering are relatively few for fractional-order strict feedback systems. In this paper, a backstepping control method based on command filtering is presented for a class of fractional-order strictly feedback nonlinear systems.

Based on the radial-basis-function neural network (NN), an adaptive control method based on approximation theory was proposed to deal with nonlinear systems with uncertain parameters [24–28] or NN [29–31] approximation. The neural network control based on the backstepping control method of command filtering is used to solve nonlinear problems in uncertain systems. It can solve the nonlinear system which does not meet the matching condition and the certain functions are nonlinear [32–35]. For a class of FOCSs, an adaptive controller via the backstepping technology similar to [36, 37] was designed in [38]. However, because it is difficult to solve the fractional-order derivative of quadratic function, the adaptive backstepping control technology of integer-order nonlinear system cannot be directly applied to the fractional nonlinear system. For FOCSs, an adaptive backstepping control method was proposed in [39], and the stability of the controller was analyzed by the integer-order Lyapunov method. Therefore, for fractional-order nonlinear systems, how to design an adaptive backstepping controller via Lyapunov stability theory is an urgent problem to be solved.

According to the above discussion, an NN adaptive backstepping control method based on command filtering is proposed for FOCSs in this paper. The NN is used to deal with unknown functions in the system. The command filters are proposed to solve the problem of item explosion caused by repeated derivation of virtual controllers, and compensation signals are designed to eliminate the errors caused by the command filter. Based on NN approximation theory, an adaptive backstepping method based on command filtering is presented. The results show that this control method can adjust the tracking error to the small neighborhood of origin and ensure that all the closed-loop system signals have the limit boundedness of half blade uniformity.

The main advantages of this approach over current results can be summarized as follows:

- The command filtered adaptive NN backstepping control can solve two problems of classical backstepping of a class of fractional-order nonlinear systems and reduce the calculation burden.
- An error compensation mechanism is introduced to overcome the shortcomings of the dynamic surface method, and the tracking error is controlled in the small neighborhood of zero.

The rest of this article is organized as follows: Section 2 is about the fuzzy logic system, fractional calculus description, and the fractional-order nonlinear systems. In Section 3, detailed controller design and stability analysis are given. In Section 4, simulation results are given to verify the effectiveness of the backstepping adaptive control method based on command filtering. Finally, Section 5 is the conclusion of this paper.

## 2. Preliminaries and Problem Description

### 2.1. Preliminaries of Fractional-Order Calculus

In recent several decades, the relatively common two kinds of fractional-order calculus are Riemann–Liouville (RL) fractional-order calculus and Caputo fractional-order calculus. The nice property of the Caputo fractional-order differential equation is that its integral value at zero makes sense. Therefore, this paper will adopt the definition of Caputo fractional-order calculus.

The fractional-order calculus operators can be regarded as a broader concept of integral calculus operators.

Thus, the definition of the fractional-order integral can be expressed as [1]

(1) I0Ctβxt=1Γβ∫0txζt−ζ1−βdζ,

where Γ(·) is the gamma function and Γ(*s*)=∫_0_^+*∞*^*t*^*s*−1^*e*^−*t*^d*t*. Consequently, the fractional-order derivative operator is represented by [1]

(2) D0Ctβxt=1Γn−β∫0txnζt−ζβ+1−ndζ.

The Laplace transform of (2) can be expressed as

(3) LD0Ctβxt=sβXs−∑k=0n−1sβ−k−1xk0,

where *X*(*s*) is the Laplace transform of *X*(*t*).

In the following sections, we examine only the case of *β* ∈ (0,1).

**Definition 1 (see [1]).**

The Mittag–Leffler function can be expressed as

(4) $$\begin{array}{c} E_{\beta ,\gamma }\left(z\right)=\sum_{k=0}^{+\infty }\frac{z^{k}}{\Gamma \left(k\beta +\gamma \right)}, \end{array}$$

with *β*, *γ* > 0, and *z* ∈ *C*. The Laplace transform of (5) can be described as

(5) $$\begin{array}{c} L\left\{t^{\gamma -1}E_{\beta ,\gamma }\left(-at^{\beta }\right)\right\}=\frac{s^{\beta -\gamma }}{s^{\beta }+a}. \end{array}$$

**Lemma 1 (see [1]).**

If there is a complex number *γ* and two real numbers *β* (0 < *β* < 1) and *ϖ*, where *ϖ* satisfies the following condition:

(6) $$\begin{array}{c} \frac{\pi \beta }{2}<\varpi <\min\left\{\pi ,\pi \beta \right\}, \end{array}$$

then, the following formula is true for all integers *n* ≥ 1:

(7) $$\begin{array}{c} E_{\beta ,\gamma }\left(z\right)=\sum_{k=0}^{n}\frac{1}{\Gamma \left(\gamma -\beta j\right)z^{j}}+o\left(\frac{1}{{\left|z\right|}^{n+1}}\right), \end{array}$$

when |*z*|⟶*∞* and *ϖ* ≤ |arg(*z*)| ≤ *n*.

**Lemma 2 (see [1]).**

If *β* (0 < *β* < 2) and *γ* are two arbitrary real numbers, and there exists a positive constant ϱ satisfying the following condition:

(8) $$\begin{array}{c} \left(\frac{v\pi }{2}\right)<\varrho \leq \min\left\{\pi ,\beta \pi \right\}, \end{array}$$

then the following inequality holds:

(9) $$\begin{array}{c} \left|E_{\beta ,\gamma }\left(z\right)\right|\leq \frac{C}{1+\left|z\right|}, \end{array}$$

where *C* > 0, ϱ ≤ |arg(*z*)| ≤ *π*, and |*z*| ≥ 0.

**Definition 2 (see [7]).**

Suppose *w* : [0, *c*)⟶ℐ is a continuous function. If the continuous function is strictly increasing and *w*(0)=0, then it is said to be class K.

**Lemma 3 (see [7]).**

If an equilibrium point of fractional-order nonlinear system *𝒟*_*t*_^*β*^*y*(*t*)=*g*(*t*, *t*(*x*)) is origin, which *g* : ℐ × Ω⟶ℛ is Lipschitz continuous. There exist a Lyapunov function *V*(*t*, *y*(*t*)) and class K functions *w*_*i*_(*i*=1,2,3) to satisfy

(10) $$\begin{array}{c} w_{1}\left(\left\|y\left(t\right)\right\|\right)\leq V\left(t,y\left(t\right)\right)\leq w_{2}\left(\left\|y\left(t\right)\right\|\right),\\ D_{t}^{\beta }V\left(t,y\left(t\right)\right)\leq w_{3}\left(\left\|y\left(t\right)\right\|\right). \end{array}$$

Then, *𝒟*_*t*_^*β*^*y*(*t*)=*g*(*t*, *t*(*x*)) is asymptotically stable; i.e., lim_*t*⟶*∞*_*y*(*t*)=0.

**Lemma 4 (see [2]).**

Assume that *y*(*t*) ∈ *𝒞*^1^([*t*_0_, *∞*], ℛ^*n*^), then

(11) $$\begin{array}{c} \frac{1}{2}D_{t}^{\beta }\left(y^{T}\left(t\right)y\left(t\right)\right)\leq y^{T}\left(t\right)D_{t}^{\beta }y\left(t\right),\;\left(\forall t\in ℐ\right). \end{array}$$

### 2.2. Preliminaries of Radial-Basis-Function NN

Let *h*(*χ*(*t*)) be a continuous unknown nonlinear function defined over a compact set Ω. Then, there exists a radial-basis-function NN to appropriate the unknown nonlinear function *h*(*χ*(*t*)) as follows:

(12) $$\begin{array}{c} \hat{h}\left(\chi \left(t\right)\right)=\theta^{T}\psi \left(\chi \left(t\right)\right), \end{array}$$

where $\hat{h}:{ℛ}^{n}⟶ℛ$ (*n* ∈ *N*) is a continuous mapping, *χ*(*t*)=[*χ*_1_(*t*), *χ*_2_(*t*),…,*χ*_*n*_(*t*)]^*T*^ is the NN input vector, *t* ≥ 0, *χ*_*i*_(*t*) ∈ *𝒞*^1^[ℐ, Ω] (*i*=1,2,…, *n*), *θ*=[*θ*_1_, *θ*_2_,…,*θ*_*N*_]^*T*^ ∈ ℛ^*N*^ (*N* > 1) is the weight vector, *ψ*(*χ*(*t*))=[*ψ*_1_(*χ*(*t*)), *ψ*_2_(*χ*(*t*)),…,*ψ*_*N*_(*χ*(*t*))]^*T*^ ∈ ℛ^*N*^ is a regressor, and *N* is the number of NN nodes. The regression variable *ψ*_*ı*_(*χ*(*t*)) selected in this paper is the Gaussian radial-basis-function

(13) $$\begin{array}{c} \psi_{ı}\left(\chi \left(t\right)\right)=\exp\left(\frac{-{\left\|x-\delta_{i}\right\|}^{2}}{2\iota_{i}^{2}}\right), \end{array}$$

where *ı*=1,2,…, *N*, *δ*_*ı*_=[*δ*_1*ı*_, *δ*_2*ı*_,…,*δ*_*nı*_]^*T*^ ∈ *R*^*n*^, *δ*_*𝒥ı*_= (*𝒥*=1,2,…, *n*) represent the centers of the Gaussian function, and *ι*_*ı*_ ∈ *R*^+^ represent the widths of the Gaussian function.

Therefore, according to the above notation, the optimal estimate of *h*(*χ*(*t*)) can be expressed as

(14) $$\begin{array}{c} h\left(\chi \left(t\right)\right)=\theta^{*T}\psi \left(\chi \left(t\right)\right)+\epsilon \left(\chi \left(t\right)\right), \end{array}$$

in which *ϵ*(*χ*(*t*))=[*ϵ*_1_(*χ*_1_(*t*)), *ϵ*_2_(*χ*_2_(*t*)),…, *ϵ*_*N*_(*χ*_*N*_(*t*))] and *θ*^**∗**^ is the optimal constant weight vector and satisfies the following condition:

(15) $$\begin{array}{c} \theta^{*}\left(t\right)=\arg\underset{\theta }{\min}\left[\text{sup}\chi \left(t\right)\left|h\left(\chi \left(t\right)\right)-\hat{h}\left(\chi \left(t\right)\right)\right|\right]. \end{array}$$

Let

(16) $$\begin{array}{c} \tilde{\theta }=\theta -\theta^{*}, \end{array}$$

in which $\tilde{\theta }$ is the parameter estimation error. According to the properties of the radial-basis-function NN, it is assumed that the optimal approximation error remains bounded. For any $\bar{\epsilon }$, there exists a sufficient number of NN nodes such that

(17) $$\begin{array}{c} \left|\epsilon_{ı}\left(\chi_{ı}\left(t\right)\right)\right|\leq {\bar{\epsilon }}_{ı}, \end{array}$$

where ${\bar{\epsilon }}_{ı}$ is a known positive constant and *ı*=1,2,…, *N*.

Then, one can obtain

(18) $$\begin{array}{c} {\hat{h}}_{ı}\left(\chi_{ı}\left(t\right),\theta_{ı}\right)-h_{ı}\left(\chi_{ı}\left(t\right)\right)\\ ={\hat{h}}_{ı}\left(\chi_{ı}\left(t\right),\theta_{ı}\right)-{\hat{h}}_{ı}\left(\chi_{ı}\left(t\right),\theta_{ı}^{*}\right)+{\hat{h}}_{ı}\left(\chi_{ı}\left(t\right),\theta_{ı}^{*}\right)-h_{ı}\left(\chi_{ı}\left(t\right)\right)\\ =\theta_{ı}^{T}\psi_{ı}\left(\chi_{ı}\left(t\right)\right)-\theta_{ı}^{*T}\psi_{ı}\left(\chi_{ı}\left(t\right)\right)+\epsilon_{ı}\left(\chi_{ı}\left(t\right)\right)\\ ={\tilde{\theta }}_{ı}^{T}\psi_{ı}\left(\chi_{ı}\left(t\right)\right)+\epsilon_{ı}\left(\chi_{ı}\left(t\right)\right), \end{array}$$

in which *ı*=1,2,…, *N*.

### 2.3. FOCSs

Consider the following FOCSs:

(19) $$\begin{array}{c} \left\{\begin{array}{c} D_{t}^{\beta }x_{1}\left(t\right)=x_{2}\left(t\right)+f_{1}\left(\bar{x_{1}}\right),\\ D_{t}^{\beta }x_{i}\left(t\right)=x_{i+1}\left(t\right)+f_{i}\left(\bar{x_{i}}\right),\\ D_{t}^{\beta }x_{n}\left(t\right)=f_{n}\left(x\left(t\right)\right)+d\left(t\right)+u\left(t\right),\\ y\left(t\right)=x_{1}\left(t\right), \end{array}\right) \end{array}$$

where *β*(0 < *β* < 1) is the system commensurate order, **x**(*t*)=[_1_(*t*), *x*_2_(*t*),…,*x*_*n*_(*t*)]^*T*^ ∈ ℛ^*n*^ is the state vector of the system, $\bar{x}\left(t\right)={\left[x_{1}\left(t\right),x_{2}\left(t\right),\ldots ,x_{i}\left(t\right)\right]}^{T}\in {ℛ}^{i}$, $f_{i}\left({\bar{x}}_{i}\right)\in ℛ\left(i=1,2,\ldots ,n\right)$ are unknown smooth nonlinear functions, *d*(*t*) ∈ ℛ is an unknown external disturbance, and *u*(*t*) ∈ ℛ and *y*(*t*) ∈ ℛ are control input and control output of the fractional-order strictly feedback nonlinear systems.

**Assumption 1 .**

For the FOCSs, if *d*(*t*) is bounded and unknown, then there exists an unknown constant $\bar{d}$ ($\bar{d}>0$) such that $\left|d\left(t\right)\right|\leq \bar{d}$ for all *t* ≥ 0.

## 3. Adaptive NN Backstepping Control Design

Let

(20) $$\begin{array}{c} s_{1}\left(t\right)=x_{1}\left(t\right)-x_{d}, \end{array}$$

(21) $$\begin{array}{c} s_{i}\left(t\right)=x_{i}\left(t\right)-x_{i,c}, \end{array}$$

in which *x*_*d*_ is a known reference signal and *x*_*i*,*c*_ (*i*=2,3,…, *n*) are the command filters.

The command filtering mechanism is defined as follows:

(22) $$\begin{array}{c} D_{t}^{\beta }x_{i+1,c}\left(t\right)=-\kappa_{i}\left(x_{i+1,c}\left(t\right)-\alpha_{i}\left(t\right)\right), \end{array}$$

where *κ*_*i*_ is a positive constant and *α*_*i*_(*t*) and *x*_*i*+1,*c*_(*t*) (*i*=1,2,…, *n* − 1) are the input signal and the output signal in the command filtering mechanism, respectively.

It is worth noting that command filtering produces errors that add to the burden of getting better tracking results. To solve this problem, an error compensation mechanism is presented to overcome the errors (*x*_*i*+1,*c*_ − *α*_*i*_) generated during the filtering process.

Then, the error compensation mechanism is defined as

(23) $$\begin{array}{c} D_{t}^{\beta }\lambda_{1}\left(t\right)=-k_{11}\lambda_{1}\left(t\right)+\lambda_{2}+x_{2,c}-\alpha_{1},\\ D_{t}^{\beta }\lambda_{i}\left(t\right)=-k_{1i}\lambda_{i}\left(t\right)-\lambda_{i-1}+\lambda_{i+1}+x_{i+1,c}-\alpha_{i},\\ D_{t}^{\beta }\lambda_{n}\left(t\right)=-k_{1n}\lambda_{n}\left(t\right)-\lambda_{n-1}, \end{array}$$

where *k*_1*i*_ > 0 are design parameters and *λ*_*i*_ (*i*=1,2,…, *n* − 1) are the error compensating signals.

The compensated tracking error signals *e*_*i*_(*t*) are designed as

(24) $$\begin{array}{c} e_{i}\left(t\right)=s_{i}\left(t\right)-\lambda_{i}\left(t\right), \end{array}$$

in which *i*=1,2,…, *n*.

Our goal is to design the control input *u* of the nonlinear systems such that the control output *y*(*t*) of the system to track *x*_*d*_ and the compensated tracking error of the nonlinear systems *e*_1_(*t*) eventually converge to a sufficiently small region of origin.

Then, the virtual control functions *α*_*i*_ (*i*=1,2,…, *n*) are constructed as

(25) $$\begin{array}{c} \alpha_{1}=-\theta_{1}^{T}\psi_{1}\left({\bar{x}}_{1}\left(t\right)\right)-k_{11}s_{1}\left(t\right)-k_{21}\text{sign}\left(e_{1}\left(t\right)\right)+D_{t}^{\beta }x_{d}\left(t\right), \end{array}$$

(26) $$\begin{array}{c} \alpha_{2}=-\theta_{2}^{T}\psi_{2}\left({\bar{x}}_{2}\left(t\right)\right)-k_{12}s_{2}\left(t\right)-k_{22}\text{sign}\left(e_{2}\left(t\right)\right)-s_{1}\left(t\right)+D_{t}^{\beta }x_{2,c}\left(t\right), \end{array}$$

(27) $$\begin{array}{c} \alpha_{i}=-\theta_{i}^{T}\psi_{i}\left({\bar{x}}_{i}\left(t\right)\right)-k_{1i}s_{i}\left(t\right)-k_{2i}\text{sign}\left(e_{i}\left(t\right)\right)-s_{i-1}\left(t\right)+D_{t}^{\beta }x_{i,c}\left(t\right), \end{array}$$

where $k_{2i}>{\bar{\epsilon }}_{i}$ (*i*=1,2,…, *n* − 1) are design parameters and ${\bar{\epsilon }}_{i}$ will be determined later.

The controller *u*(*t*) is designed as follows:

(28) $$\begin{array}{c} u\left(t\right)=-\theta_{n}^{T}\psi_{n}\left(x_{n}\left(t\right)\right)-k_{1n}s_{n}\left(t\right)-s_{n-1}\left(t\right)-\left(k_{2n}+\hat{\bar{d}}\left(t\right)\right)\text{sign}\left(e_{n}\left(t\right)\right)+D_{t}^{\beta }x_{n,c}\left(t\right), \end{array}$$

in which $\hat{\bar{d}}\left(t\right)$ is the estimation of $\bar{d}$, $k_{2n}>{\bar{\epsilon }}_{n}$ is a design parameter, and ${\bar{\epsilon }}_{n}$ will be determined later.

Then, the backstepping control algorithms of the fractional-order nonlinear system are shown as follows.

**Step 1 .**

Consider the first Lyapunov function *v*_1_(*x*) as follows:

(29) $$\begin{array}{c} v_{1}\left(t\right)=\frac{1}{2}e_{1}^{2}\left(t\right). \end{array}$$

According to Lemma 4, differentiating *v*_1_(*x*) can gain

(30) $$\begin{array}{c} D_{t}^{\beta }v_{1}\left(t\right)\leq e_{1}\left(t\right)D_{t}^{\beta }e_{1}\left(t\right)\\ =e_{1}\left(t\right)\left(D_{t}^{\beta }x_{1}\left(t\right)-D_{t}^{\beta }x_{d}\left(t\right)-D_{t}^{\beta }\lambda_{1}\left(t\right)\right)\\ =e_{1}\left(t\right)\left[x_{2}\left(t\right)+f_{1}\left({\bar{x}}_{1}\left(t\right)\right)-D_{t}^{\beta }x_{d}\left(t\right)-D_{t}^{\beta }\lambda_{1}\left(t\right)\right]\\ =e_{1}\left(t\right)\left[s_{2}\left(t\right)+x_{2,c}\left(t\right)+f_{1}\left({\bar{x}}_{1}\left(t\right)\right)-{\hat{f}}_{1}\left({\bar{x}}_{1}\left(t,\theta_{1}^{*}\right)\right)+{\hat{f}}_{1}\left({\bar{x}}_{1}\left(t,\theta_{1}^{*}\right)\right)\right.\\ -D_{t}^{\beta }x_{d}\left(t\right)+k_{11}\lambda_{1}\left(t\right)-\lambda_{2}\left(t\right)-x_{2,c}\left(t\right)+\beta_{1}\left(t\right)\\ =e_{1}\left(t\right)\left[e_{2}\left(t\right)-{\tilde{\theta }}_{1}^{T}\psi_{1}\left({\bar{x}}_{1}\left(t\right)\right)-\epsilon_{1}\left({\bar{x}}_{1}\left(t\right)\right)+\theta_{1}^{T}\psi_{1}\left({\bar{x}}_{1}\left(t\right)\right)\right)\\ -D_{t}^{\beta }x_{d}\left(t\right)+k_{11}\lambda_{1}\left(t\right)+\alpha_{1}\left(t\right), \end{array}$$

in which $\epsilon_{1}\left({\bar{x}}_{1}\left(t\right)\right)={\hat{f}}_{1}\left({\bar{x}}_{1}\left(t,\theta^{*}\right)\right)-f_{1}\left({\bar{x}}_{1}\left(t\right)\right)$ and ${\tilde{\theta }}_{1}=\theta_{1}-\theta_{1}^{*}$.

Substituting (25) into (30), we can obtain

(31) $$\begin{array}{c} D_{t}^{\beta }v_{1}\left(t\right)\leq e_{1}\left(t\right)\left[e_{2}\left(t\right)-k_{11}e_{1}\left(t\right)-{\tilde{\theta }}_{1}^{T}\psi_{1}\left({\bar{x}}_{1}\left(t\right)\right)-\epsilon_{1}\left({\bar{x}}_{1}\left(t\right)\right)-k_{21}\text{sign}\left(e_{1}\left(t\right)\right)\right]\\ \leq e_{1}\left(t\right)e_{2}\left(t\right)-k_{11}e_{1}^{2}\left(t\right)-e_{1}\left(t\right){\tilde{\theta }}_{1}^{T}\psi_{1}\left({\bar{x}}_{1}\left(t\right)\right)+{\bar{\epsilon }}_{1}\left|e_{1}\left(t\right)\right|-k_{21}\left|e_{1}\left(t\right)\right|\\ \leq e_{1}\left(t\right)e_{2}\left(t\right)-k_{11}e_{1}^{2}\left(t\right)-e_{1}\left(t\right){\tilde{\theta }}_{1}^{T}\psi_{1}\left({\bar{x}}_{1}\left(t\right)\right), \end{array}$$

where *k*_11_ > 0 and $k_{21}>{\bar{\epsilon }}_{1}$ are design parameters.

Choose the Lyapunov function *V*_1_(*x*) as follows:

(32) $$\begin{array}{c} V_{1}\left(t\right)=v_{1}\left(t\right)+\frac{1}{2\rho_{1}}{\tilde{\theta }}_{1}^{T}\left(t\right){\tilde{\theta }}_{1}\left(t\right). \end{array}$$

The design of adaptive law is as follows:

(33) $$\begin{array}{c} D_{t}^{\beta }\theta_{1}=\rho_{1}e_{1}\left(t\right)\psi_{1}\left({\bar{x}}_{1}\left(t\right)\right)-\gamma_{1}\theta_{1}, \end{array}$$

where *ρ*_1_ and *γ*_1_ are positive design parameters.

Because *θ*_1_^*∗*^ is a constant, then we have

(34) $$\begin{array}{c} D_{t}^{\beta }{\tilde{\theta }}_{1}=D_{t}^{\beta }\theta_{1}. \end{array}$$

Then, according to Lemma 4, we can have

(35) $$\begin{array}{c} D_{t}^{\beta }V_{1}\left(t\right)\leq D_{t}^{\beta }v_{1}\left(t\right)+\frac{1}{\rho_{1}}{\tilde{\theta }}_{1}^{T}D_{t}^{\beta }{\tilde{\theta }}_{1}. \end{array}$$

Substituting formulas (31) and (33) into the above inequality, the following can be obtained:

(36) $$\begin{array}{c} D_{t}^{\beta }V_{1}\left(t\right)\leq -k_{11}e_{1}^{2}\left(t\right)+e_{1}\left(t\right)e_{2}\left(t\right)-\frac{1}{\rho_{1}}{\tilde{\theta }}_{1}^{T}\theta_{1}\\ \leq -k_{11}e_{1}^{2}\left(t\right)+e_{1}\left(t\right)e_{2}\left(t\right)-\frac{1}{\rho_{1}}{\tilde{\theta }}_{1}^{T}{\tilde{\theta }}_{1}-\frac{1}{\rho_{1}}{\tilde{\theta }}_{1}^{T}\theta_{1}^{*}. \end{array}$$

According to Young's inequality, then we can obtain

(37) $$\begin{array}{c} -\frac{1}{\rho_{1}}{\tilde{\theta }}_{1}^{T}\theta_{1}^{*}\leq \frac{1}{2\rho_{1}}\theta_{1}^{*T}\theta_{1}^{*}+\frac{1}{2\rho_{1}}{\tilde{\theta }}_{1}^{T}{\tilde{\theta }}_{1}. \end{array}$$

According to formulas (36) and (37), it can be obtained

(38) $$\begin{array}{c} D_{t}^{\beta }V_{1}\left(t\right)\leq -k_{11}e_{1}^{2}\left(t\right)+e_{1}\left(t\right)e_{2}\left(t\right)-\frac{1}{2\rho_{1}}{\tilde{\theta }}_{1}^{T}{\tilde{\theta }}_{1}+\frac{1}{2\rho_{1}}\theta_{1}^{*T}\theta_{1}^{*}\\ \leq -k_{1}V_{1}\left(t\right)+e_{1}\left(t\right)e_{2}\left(t\right)+\Theta_{1}, \end{array}$$

in which *k*_1_=min{2*k*_11_, *γ*_1_} and Θ_1_=(*γ*_1_/2*ρ*_1_)*θ*_1_^*∗T*^*θ*_1_^*∗*^ are two positive constants.

**Step 2 .**

Consider the second Lyapunov function *v*_2_(*x*) as follows:

(39) $$\begin{array}{c} v_{2}\left(t\right)=\frac{1}{2}e_{2}^{2}\left(t\right). \end{array}$$

According to Lemma 4, differentiating *v*_2_(*x*) can gain

(40) $$\begin{array}{c} D_{t}^{\beta }v_{2}\left(t\right)\leq e_{2}\left(t\right)D_{t}^{\beta }e_{2}\left(t\right)\\ =e_{2}\left(t\right)\left(D_{t}^{\beta }x_{2}\left(t\right)-D_{t}^{\beta }x_{2,c}\left(t\right)-D_{t}^{\beta }\lambda_{2}\left(t\right)\right)\\ =e_{2}\left(t\right)\left[x_{3}\left(t\right)+f_{2}{\bar{x}}_{2}\left(t\right)-D_{t}^{\beta }x_{2,c}\left(t\right)-D_{t}^{\beta }\lambda_{2}\left(t\right)\right]\\ =e_{2}\left(t\right)s_{3}\left(t\right)+x_{3,c}\left(t\right)+f_{2}\left({\bar{x}}_{2}\left(t\right)\right)-{\hat{f}}_{2}\left({\bar{x}}_{2}\left(t,\theta_{2}^{*}\right)\right)+{\hat{f}}_{2}\left({\bar{x}}_{2}\left(t,\theta_{2}^{*}\right)\right)\\ -D_{t}^{\beta }x_{2,c}\left(t\right)+k_{12}\lambda_{2}+\lambda_{1}\left(t\right)-\lambda_{3}\left(t\right)-x_{3,c}\left(t\right)+\alpha_{2}\left(t\right)\\ =e_{2}\left(t\right)e_{3}\left(t\right)-{\tilde{\theta }}_{2}^{T}\psi_{2}\left({\bar{x}}_{2}\left(t\right)\right)-\epsilon_{2}\left({\bar{x}}_{2}\left(t\right)\right)+\theta_{2}^{T}\psi_{2}\left({\bar{x}}_{2}\left(t\right)\right)-D_{t}^{\beta }x_{2,c}\left(t\right)\\ +k_{12}\lambda_{2}+\lambda_{1}\left(t\right)+\alpha_{2}\left(t\right), \end{array}$$

in which $\epsilon_{2}\left({\bar{x}}_{2}\left(t\right)\right)={\hat{f}}_{2}\left({\bar{x}}_{2}\left(t,\theta^{*}\right)\right)-f_{2}\left({\bar{x}}_{2}\left(t\right)\right)$ and${\tilde{\theta }}_{2}=\theta_{2}-\theta_{2}^{*}$.

Substituting (27) into (41), we can obtain

(41) $$\begin{array}{c} D_{t}^{\beta }v_{2}\left(t\right)\leq e_{2}\left(t\right)\left[e_{3}\left(t\right)-k_{12}e_{2}\left(t\right)-{\tilde{\theta }}_{2}^{T}\psi_{2}\left({\bar{x}}_{2}\left(t\right)\right)-\epsilon_{2}\left({\bar{x}}_{2}\left(t\right)\right)-k_{22}\text{sign}\left(e_{2}\left(t\right)\right)-e\left(t\right)\right]\\ \leq e_{2}\left(t\right)e_{3}\left(t\right)-k_{12}e_{2}^{2}\left(t\right)-e_{2}\left(t\right){\tilde{\theta }}_{2}^{T}\psi_{2}\left({\bar{x}}_{2}\left(t\right)\right)+{\bar{\epsilon }}_{2}\left|e_{2}\left(t\right)\right|\\ -k_{22}\left|e_{2}\left(t\right)\right|-e_{1}\left(t\right)e_{2}\left(t\right)\\ \leq -k_{12}e_{2}^{2}+e_{2}\left(t\right)e_{3}\left(t\right)-e_{2}\left(t\right){\tilde{\theta }}_{2}^{T}\psi_{2}\left({\bar{x}}_{2}\left(t\right)\right)-e_{1}\left(t\right)e_{2}\left(t\right), \end{array}$$

where *k*_12_ > 0 and $k_{22}>{\bar{\varepsilon }}_{2}$ are design parameters.

Choose the Lyapunov function *V*_2_(*x*) as follows:

(42) $$\begin{array}{c} V_{2}\left(t\right)=V_{1}\left(t\right)+v_{2}\left(t\right)+\frac{1}{2\rho_{2}}{\tilde{\theta }}_{2}^{T}\left(t\right){\tilde{\theta }}_{2}\left(t\right). \end{array}$$

The design of adaptive law is as follows:

(43) $$\begin{array}{c} D_{t}^{\beta }\theta_{2}=\rho_{2}e_{2}\left(t\right)\psi_{2}\left({\bar{x}}_{2}\left(t\right)\right)-\gamma_{2}\theta_{2}, \end{array}$$

where *ρ*_2_ and *γ*_2_ are positive design parameters.

Because *θ*_2_^*∗*^ is a constant, then we have

(44) $$\begin{array}{c} D_{t}^{\beta }{\tilde{\theta }}_{2}=D_{t}^{\beta }\theta_{2}. \end{array}$$

Then, according to Lemma 4 and formulas (42) and (44), we can have

(45) $$\begin{array}{c} D_{t}^{\beta }V_{2}\left(t\right)\leq -kV_{1}\left(t\right)+e_{1}\left(t\right)e_{2}\left(t\right)+\Theta_{1}+D_{t}^{\beta }v_{2}\left(t\right)+\frac{1}{\rho_{2}}{\tilde{\theta }}_{2}^{T}D_{t}^{\beta }{\tilde{\theta }}_{2}. \end{array}$$

According to formulas (45) and (46), the above equation can be reduced to

(46) $$\begin{array}{c} D_{t}^{\beta }V_{2}\left(t\right)\leq -k_{1}V_{1}\left(t\right)+\Theta_{1}-k_{12}e_{2}^{2}\left(t\right)+e_{2}\left(t\right)e_{3}\left(t\right)-\frac{1}{2\rho_{2}}{\tilde{\theta }}_{2}^{T}{\tilde{\theta }}_{2}+\frac{1}{2\rho_{2}}\theta_{2}^{*T}\theta_{2}^{*}\\ \leq -k_{2}V_{2}\left(t\right)+e_{2}\left(t\right)e_{3}\left(t\right)+\Theta_{2}, \end{array}$$

in which *k*_2_=min{*k*_1_, 2*k*_12_, *γ*_2_} and Θ_2_=Θ_1_+(*γ*_2_/2*ρ*_2_)*θ*_2_^*∗T*^*θ*_2_^*∗*^ are two positive constants.

**Step 3 .**

*i*  (*i*=3,4,…, *n* − 1).

Consider the *i*th Lyapunov function *v*_*i*_(*x*) as follows:

(47) $$\begin{array}{c} v_{i}\left(t\right)=\frac{1}{2}e_{i}^{2}\left(t\right). \end{array}$$

According to Lemma 4, differentiating *v*_*i*_(*x*) can gain

(48) $$\begin{array}{c} D_{t}^{\beta }v_{i}\left(t\right)\leq e_{i}\left(t\right)D_{t}^{\beta }e_{i}\left(t\right)\\ =e_{i}\left(t\right)\left(D_{t}^{\beta }x_{i}\left(t\right)-D_{t}^{\beta }x_{i,c}\left(t\right)-D_{t}^{\beta }\lambda_{i}\left(t\right)\right)\\ =e_{i}\left(t\right)\left[x_{i+1}\left(t\right)+f_{i}\left({\bar{x}}_{i}\left(t\right)\right)-D_{t}^{\beta }x_{i,c}\left(t\right)-D_{t}^{\beta }\lambda_{i}\left(t\right)\right]\\ =e_{i}\left(t\right)s_{i+1}\left(t\right)+x_{i+1,c}\left(t\right)+f_{i}\left({\bar{x}}_{i}\left(t\right)\right)-{\hat{f}}_{i}\left({\bar{x}}_{i}\left(t,\theta_{i}^{*}\right)\right)+{\hat{f}}_{i}\left({\bar{x}}_{i}\left(t,\theta_{i}^{*}\right)\right)\\ -D_{t}^{\beta }x_{i,c}\left(t\right)+k_{1i}\lambda_{i}+\lambda_{i-1}\left(t\right)-\lambda_{i+1}\left(t\right)-x_{i+1,c}\left(t\right)+\alpha_{i}\left(t\right)\\ =e_{i}\left(t\right)e_{i+1}\left(t\right)-{\tilde{\theta }}_{i}^{T}\psi_{i}\left({\bar{x}}_{i}\left(t\right)\right)-\epsilon_{i}\left({\bar{x}}_{i}\left(t\right)\right)+\theta_{i}^{T}\psi_{i}\left({\bar{x}}_{i}\left(t\right)\right)\\ -D_{t}^{\beta }x_{i,c}\left(t\right)+k_{1i}\lambda_{i}+\lambda_{i-1}\left(t\right)+\alpha_{i}\left(t\right), \end{array}$$

in which $\epsilon_{i}\left({\bar{x}}_{i}\left(t\right)\right)={\hat{f}}_{i}\left({\bar{x}}_{i}\left(t,\theta^{*}\right)\right)-f_{i}\left({\bar{x}}_{i}\left(t\right)\right)$ and ${\tilde{\theta }}_{i}=\theta_{i}-\theta_{i}^{*}$.

Substituting (27) into (49), we can obtain

(49) $$\begin{array}{c} D_{t}^{\beta }v_{i}\left(t\right)\leq e_{i}\left(t\right)\left[e_{i+1}\left(t\right)-k_{1i}e_{i}\left(t\right)-{\tilde{\theta }}_{i}^{T}\psi_{i}\left({\bar{x}}_{i}\left(t\right)\right)-\epsilon_{i}\left({\bar{x}}_{i}\left(t\right)\right)-k_{2i}\text{sign}\left(e_{i}\left(t\right)\right)-e_{i-1}\left(t\right)\right]\\ \leq e_{i}\left(t\right)e_{i+1}\left(t\right)-k_{1i}e_{i}^{2}\left(t\right)-e_{i}\left(t\right){\tilde{\theta }}_{i}^{T}\psi_{i}\left({\bar{x}}_{i}\left(t\right)\right)+{\bar{\epsilon }}_{i}\left|e_{i}\left(t\right)\right|-k_{2i}\left|e_{i}\left(t\right)\right|-e_{i-1}\left(t\right)e_{i}\left(t\right)\\ \leq e_{i}\left(t\right)e_{i+1}\left(t\right)-k_{1i}e_{i}^{2}-e_{i}\left(t\right){\tilde{\theta }}_{i}^{T}\psi_{i}\left({\bar{x}}_{i}\left(t\right)\right)-e_{i-1}\left(t\right)e_{i}\left(t\right), \end{array}$$

where *k*_1*i*_ > 0 and $k_{2i}>{\bar{\epsilon }}_{i}$ are design parameters.

Choose the Lyapunov function *V*_*i*_(*x*) as follows:

(50) $$\begin{array}{c} V_{i}\left(t\right)=V_{i-1}\left(t\right)+v_{i}\left(t\right)+\frac{1}{2\rho_{i}}{\tilde{\theta }}_{i}^{T}\left(t\right){\tilde{\theta }}_{i}\left(t\right). \end{array}$$

The design of adaptive law is as follows:

(51) $$\begin{array}{c} D_{t}^{\beta }\theta_{i}=\rho_{i}e_{i}\left(t\right)\psi_{i}\left({\bar{x}}_{i}\left(t\right)\right)-\gamma_{i}\theta_{i}, \end{array}$$

where *ρ*_*i*_ and *γ*_*i*_ are positive design parameters.

Because *θ*_*i*_^*∗*^ is a constant, then we have

(52) $$\begin{array}{c} D_{t}^{\beta }{\tilde{\theta }}_{i}=D_{t}^{\beta }\theta_{i}. \end{array}$$

Then, according to Lemma 4, we can have

(53) $$\begin{array}{c} D_{t}^{\beta }V_{i}\left(t\right)\leq -k_{i-1}V_{i-1}\left(t\right)+e_{i-1}\left(t\right)e_{i}\left(t\right)+\Theta_{i-1}+D_{t}^{\beta }v_{i}\left(t\right)+\frac{1}{\rho_{i}}{\tilde{\theta }}_{i}^{T}D_{t}^{\beta }{\tilde{\theta }}_{i}. \end{array}$$

From formulas (50), (52), and (54), we have

(54) $$\begin{array}{c} D_{t}^{\beta }V_{i}\left(t\right)\leq -k_{i-1}V_{i-1}\left(t\right)+\Theta_{i-1}+e_{i}\left(t\right)e_{i+1}\left(t\right)-k_{1i}e_{i}^{2}-\frac{1}{\rho_{i}}{\tilde{\theta }}_{i}^{T}\theta_{i}\\ \leq -k_{i-1}V_{i-1}\left(t\right)+\Theta_{i-1}-k_{1i}e_{i}^{2}\left(t\right)+e_{i}\left(t\right)e_{i+1}\left(t\right)-\frac{1}{\rho_{i}}{\tilde{\theta }}_{i}^{T}{\tilde{\theta }}_{i}-\frac{1}{\rho_{i}}{\tilde{\theta }}_{i}^{T}\theta_{i}^{*}. \end{array}$$

According to Young's inequality, then we can obtain

(55) $$\begin{array}{c} -\frac{1}{\rho_{i}}{\tilde{\theta }}_{i}^{T}\theta_{i}^{*}\leq \frac{1}{2\rho_{i}}\theta_{i}^{*T}\theta_{i}^{*}+\frac{1}{2\rho_{i}}{\tilde{\theta }}_{i}^{T}{\tilde{\theta }}_{i}. \end{array}$$

Substitute the above formula into (55), the following can be obtained

(56) $$\begin{array}{c} D_{t}^{\beta }V_{i}\left(t\right)\leq -k_{i-1}V_{i-1}\left(t\right)+\Theta_{i-1}-k_{1i}e_{i}^{2}\left(t\right)+e_{i}\left(t\right)e_{i+1}\left(t\right)-\frac{1}{2\rho_{i}}{\tilde{\theta }}_{i}^{T}{\tilde{\theta }}_{i}+\frac{1}{2\rho_{i}}\theta_{i}^{*T}\theta_{i}^{*}\\ \leq -k_{i}V_{i}\left(t\right)+e_{i+1}\left(t\right)e_{i}\left(t\right)+\Theta_{i}, \end{array}$$

in which *k*_*i*_=min{*k*_*i*−1_, 2*k*_1*i*_, *γ*_*i*_} and Θ_*i*_=Θ_*i*−1_+(*γ*_*i*_/2*ρ*_*i*_)*θ*_*i*_^*∗T*^*θ*_*i*_^*∗*^ are two positive constants.

**Step 4 .**

*n*.

Consider the *n*th Lyapunov function *v*_*n*_(*x*) as follows:

(57) $$\begin{array}{c} v_{n}\left(t\right)=\frac{1}{2}e_{n}^{2}\left(t\right). \end{array}$$

According to Lemma 4, differentiating *v*_*n*_(*x*) can gain

(58) $$\begin{array}{c} D_{t}^{\beta }v_{n}\left(t\right)\leq e_{n}\left(t\right)D_{t}^{\beta }e_{n}\left(t\right)\\ =e_{n}\left(t\right)\left(D_{t}^{\beta }x_{n}\left(t\right)-D_{t}^{\beta }x_{n,c}\left(t\right)-D_{t}^{\beta }\lambda_{n}\left(t\right)\right)\\ =e_{n}\left(t\right)\left[f_{n}\left({\bar{x}}_{n}\left(t\right)\right)+d\left(t\right)+u\left(t\right)-D_{t}^{\beta }x_{n,c}\left(t\right)-D_{t}^{\beta }\lambda_{n}\left(t\right)\right]\\ =e_{n}\left(t\right)f_{n}\left({\bar{x}}_{n}\left(t\right)\right)-{\hat{f}}_{n}\left({\bar{x}}_{n}\left(t,\theta_{n}^{*}\right)\right)+{\hat{f}}_{n}\left({\bar{x}}_{n}\left(t,\theta_{n}^{*}\right)\right)\\ +d\left(t\right)+u\left(t\right)-D_{t}^{\beta }x_{n,c}\left(t\right)+k_{1n}\lambda_{n}+\lambda_{n-1}\\ =e_{n}\left(t\right)-{\tilde{\theta }}_{n}^{T}\psi_{n}\left({\bar{x}}_{n}\left(t\right)\right)-\epsilon_{n}\left({\bar{x}}_{n}\left(t\right)\right)+\theta_{n}^{T}\psi_{n}\left({\bar{x}}_{n}\left(t\right)\right)\\ +d\left(t\right)+u\left(t\right)-D_{t}^{\beta }x_{n,c}\left(t\right)+k_{1n}\lambda_{n}+\lambda_{n-1}, \end{array}$$

in which $\epsilon_{n}\left({\bar{x}}_{n}\left(t\right)\right)={\hat{f}}_{n}\left({\bar{x}}_{n}\left(t,\theta^{*}\right)\right)-f_{n}\left({\bar{x}}_{n}\left(t\right)\right)$ and ${\tilde{\theta }}_{n}=\theta_{n}-\theta_{n}^{*}$.

Substituting (28) into (59), one obtains

(59) $$\begin{array}{c} D_{t}^{\beta }v_{n}\left(t\right)\leq e_{n}\left(t\right)\left[-k_{1n}e_{n}\left(t\right)-{\tilde{\theta }}_{n}^{T}\psi_{n}\left({\bar{x}}_{n}\left(t\right)\right)-\epsilon_{n}\left({\bar{x}}_{n}\left(t\right)\right)-\left(k_{2n}+\hat{\bar{d}}\left(t\right)\right)\text{sign}\left(e_{n}\left(t\right)\right)-e_{n-1}\left(t\right)+d\left(t\right)\right]\\ \leq -k_{1n}e_{n}^{2}\left(t\right)-e_{n}\left(t\right){\tilde{\theta }}_{n}^{T}\psi_{n}\left({\bar{x}}_{n}\left(t\right)\right)+{\bar{\epsilon }}_{n}\left|e_{n}\left(t\right)\right|\\ -\left(k_{2n}+\hat{\bar{d}}\left(t\right)\right)\left|e_{n}\left(t\right)\right|-e_{n-1}\left(t\right)e_{n}\left(t\right)+\bar{d}\left|e_{n}\left(t\right)\right|\\ \leq -k_{1n}e_{n}^{2}-e_{n}\left(t\right){\tilde{\theta }}_{n}^{T}\psi_{n}\left({\bar{x}}_{n}\left(t\right)\right)-e_{n-1}\left(t\right)e_{n}\left(t\right)-\tilde{\bar{d}}\left(t\right)\left|e_{n}\left(t\right)\right|, \end{array}$$

where *k*_1*n*_ > 0 and $k_{2n}>{\bar{\epsilon }}_{n}$ are design parameters and $\tilde{\bar{d}}\left(t\right)=\hat{\bar{d}}\left(t\right)-\bar{d}$ is the estimation error.

Choose the Lyapunov function *V*_*n*_(*x*) as follows:

(60) $$\begin{array}{c} V_{n}\left(t\right)=V_{n-1}\left(t\right)+v_{n}\left(t\right)+\frac{1}{2\rho_{n}}{\tilde{\theta }}_{n}^{T}\left(t\right){\tilde{\theta }}_{n}\left(t\right)+\frac{1}{2\xi_{1}}{\tilde{\bar{d}}}^{2}\left(t\right). \end{array}$$

The design of adaptive law is as follows:

(61) $$\begin{array}{c} D_{t}^{\beta }\theta_{n}=\rho_{n}e_{n}\left(t\right)\psi_{n}\left({\bar{x}}_{n}\left(t\right)\right)-\gamma_{n}\theta_{n}, \end{array}$$

(62) $$\begin{array}{c} D_{t}^{\beta }\hat{\bar{d}}\left(t\right)=\xi_{1}\left|e_{n}\right|-\xi_{2}\hat{\bar{d}}\left(t\right), \end{array}$$

where *ρ*_*n*_, *γ*_*n*_, *ξ*_1_, and *ξ*_2_ are positive design parameters.

Because *θ*_*n*_^*∗*^ is a constant, then we have

(63) $$\begin{array}{c} D_{t}^{\beta }{\tilde{\theta }}_{n}=D_{t}^{\beta }\theta_{n}. \end{array}$$

Then, according to Lemma 4, we can have

(64) $$\begin{array}{c} D_{t}^{\beta }V_{n}\left(t\right)\leq -k_{n-1}V_{n-1}\left(t\right)+e_{n-1}\left(t\right)e_{n}\left(t\right)+\Theta_{n-1}+D_{t}^{\beta }v_{n}\left(t\right)\\ +\frac{1}{\rho_{n}}{\tilde{\theta }}_{n}^{T}D_{t}^{\beta }{\tilde{\theta }}_{n}+\frac{1}{\xi_{1}}\tilde{\bar{d}}\left(t\right)D_{t}^{\beta }\tilde{\bar{d}}\left(t\right). \end{array}$$

From formulas (60) and (65), we have

(65) $$\begin{array}{c} D_{t}^{\beta }V_{n}\left(t\right)\leq -k_{n-1}V_{n-1}\left(t\right)+\Theta_{n-1}-k_{1n}e_{n}^{2}-e_{n}\left(t\right){\tilde{\theta }}_{n}^{T}\psi_{n}\left({\bar{x}}_{n}\left(t\right)\right)\\ -\tilde{\bar{d}}\left(t\right)\left|e_{n}\left(t\right)\right|+\frac{1}{\rho_{n}}{\tilde{\theta }}_{n}^{T}D_{t}^{\beta }{\tilde{\theta }}_{n}+\frac{1}{\xi_{1}}\tilde{\bar{d}}\left(t\right)D_{t}^{\beta }\tilde{\bar{d}}\left(t\right). \end{array}$$

By substituting (62) and (63) into the above inequality, it can be obtained

(66) $$\begin{array}{c} D_{t}^{\beta }V_{n}\left(t\right)\leq -k_{n-1}V_{n-1}\left(t\right)+\Theta_{n-1}-k_{1n}e_{n}^{2}-\frac{\gamma_{n}}{\rho_{n}}{\tilde{\theta }}_{n}^{T}\theta_{n}-\frac{\xi_{2}}{\xi_{1}}\tilde{\bar{d}}\left(t\right)\hat{\bar{d}}\left(t\right)\\ \leq -k_{n-1}V_{n-1}\left(t\right)+\Theta_{n-1}-k_{1n}e_{n}^{2}-\frac{\gamma_{n}}{\rho_{n}}{\tilde{\theta }}_{n}^{T}{\tilde{\theta }}_{n}-\frac{\gamma_{n}}{\rho_{n}}{\tilde{\theta }}_{n}^{T}\theta_{n}^{*}\\ -\frac{\xi_{2}}{\xi_{1}}{\tilde{\bar{d}}}^{2}\left(t\right)-\frac{\xi_{2}}{\xi_{1}}\tilde{\bar{d}}\left(t\right)\bar{d}. \end{array}$$

According to Young's inequality, we can obtain

(67) $$\begin{array}{c} -\frac{1}{\rho_{i}}{\tilde{\theta }}_{n}^{T}\theta_{n}^{*}\leq \frac{1}{2\rho_{n}}\theta_{n}^{*T}\theta_{n}^{*}+\frac{1}{2\rho_{n}}{\tilde{\theta }}_{n}^{T}{\tilde{\theta }}_{n}, \end{array}$$

(68) $$\begin{array}{c} -\frac{\xi_{2}}{\xi_{1}}\tilde{\bar{d}}\left(t\right)\bar{d}\left(t\right)\leq \frac{\xi_{2}}{2\xi_{1}}{\tilde{\bar{d}}}^{2}\left(t\right)+\frac{\xi_{2}}{2\xi_{1}}{\bar{d}}^{2}. \end{array}$$

Substitute the above formulas into (67), one obtains

(69) $$\begin{array}{c} D_{t}^{\beta }V_{i}\left(t\right)\leq -k_{n-1}V_{n-1}\left(t\right)+\Theta_{n-1}-k_{1n}e_{n}^{2}-\frac{\gamma_{n}}{2\rho_{n}}{\tilde{\theta }}_{n}^{T}{\tilde{\theta }}_{n}+\frac{\gamma_{n}}{2\rho_{n}}\theta_{n}^{*T}\theta_{n}^{*}\\ -\frac{\xi_{2}}{2\xi_{1}}{\tilde{\bar{d}}}^{2}\left(t\right)+\frac{\xi_{2}}{2\xi_{1}}{\bar{d}}^{2}\leq -k_{n}V_{n}\left(t\right)+\Theta_{n}, \end{array}$$

in which *k*_*n*_=min{*k*_*n*−1_, 2*k*_1*n*_, *γ*_*n*_, *ξ*_2_} and $\Theta_{n}=\Theta_{n-1}+\left(\gamma_{n}/2\rho_{n}\right)\theta_{n}^{*T}\theta_{n}^{*}+\left(\xi_{2}/2\xi_{1}\right){\bar{d}}^{2}$ are two positive constants.

The stability analysis of fractional-order nonlinear systems is shown below:

**Theorem 1 .**

Consider system (20) under Assumption 1. The control input is constructed as (29) with (26)–(28), and the adaptation laws are designed as (34), (44), (52), (62), and (63); then, there exist appropriate design parameters, such that the tracking error *e*_1_(*t*) tends to an arbitrarily small region of the origin.

**Proof**

According to (70), there exists a function *q*(*t*) ≥ 0 such that the following equation holds on:

(70) $$\begin{array}{c} D_{t}^{\beta }V_{i}\left(t\right)+q\left(t\right)=-k_{n}V_{n}\left(t\right)+\Theta_{n}. \end{array}$$

The Laplace transform of equation (71) is applied to obtain

(71) $$\begin{array}{c} V_{n}\left(s\right)=\frac{s^{\beta -1}}{s^{\beta }+k_{n}}V_{n}\left(0\right)+\frac{\Theta_{n}}{s\left(s^{\beta }+k_{n}\right)}-\frac{Q\left(s\right)}{s^{\beta }+k_{n}}\\ =\frac{s^{\beta -1}}{s^{\beta }+k_{n}}V_{n}\left(0\right)+\frac{s^{\beta -\left(1+\beta \right)}\Theta_{n}}{s_{\beta }+k_{n}}-\frac{Q\left(s\right)}{s^{\beta }+k_{n}}, \end{array}$$

in which *V*_*n*_(*s*) and *Q*(*s*) are Laplace transforms of *V*_*n*_(*t*) and *q*(*t*), respectively, and *V*_*n*_(0)is the initial condition. According to (6) and (71), it can be represented as

(72) $$\begin{array}{c} V_{n}\left(t\right)=V_{n}\left(0\right)E_{\beta ,1}\left(-k_{n}t^{\beta }\right)+\Theta_{n}t^{\beta }E_{\beta ,1+\beta }\left(-k_{n}t^{\beta }\right)-q\left(t\right)*t^{-1}E_{\beta ,0}\left(-k_{n}t^{\beta }\right), \end{array}$$

where *∗* is the convolution operator. If sine *q*(*t*) and *t*^−1^*E*_*β*,0_(−*k*_*n*_*t*^*β*^) are both nonnegative functions, then *q*(*t*)*∗t*^−1^*E*_*β*,0_(−*k*_*n*_*t*^*β*^) ≥ 0 can be obtained. Hence, the following inequality holds on:

(73) $$\begin{array}{c} \left|V_{n}\left(t\right)\right|\leq \left|V_{n}\left(0\right)\right|E_{\beta ,1}\left(-k_{n}t^{\beta }\right)+\Theta_{n}t^{\beta }E_{\beta ,1+\beta }\left(-k_{n}t^{\beta }\right). \end{array}$$

Because arg(−*k*_*n*_*t*^*β*^)=−*π*, |−*k*_*n*_*t*^*β*^| ≥ 0, for all *t* ≥ 0 and *β* ∈ (0,2) and according to Lemma 2, then there exists a positive constant *A* such that

(74) $$\begin{array}{c} \left|E_{\beta ,1}\left(-k_{n}t^{\beta }\right)\right|\leq \frac{A}{1+k_{n}t^{\beta }}. \end{array}$$

According to (75), one can obtain

(75) $$\begin{array}{c} \underset{t⟶\infty }{\text{lim}}\left|V_{n}\left(0\right)\right|E_{\beta ,1}\left(-k_{n}t^{\beta }\right)=0. \end{array}$$

Consequently, for every *η* > 0, there exists a constant *t*_*r*_ > 0, such that *t* > *t*_*r*_ holds on, then one can obtain

(76) $$\begin{array}{c} \left|V_{n}\left(0\right)\right|E_{\beta ,1}\left(-k_{n}t^{\beta }\right)<\frac{\eta }{3}. \end{array}$$

In addition, according to Lemma 1, one can obtain

(77) $$\begin{array}{c} E_{\beta ,\beta +1}\left(-k_{n}t^{\beta }\right)=\frac{1}{\Gamma \left(1\right)k_{n}t^{\beta }}+o\left(\frac{1}{{\left|-k_{n}t^{\beta }\right|}^{1+1}}\right), \end{array}$$

in which the integer *n* is chosen as 1. According to (78), for every *η* > 0, there exists a constant *t*_*b*_ > 0, such that *t* > *t*_*b*_ holds on, one obtains

(78) $$\begin{array}{c} \Theta_{n}t^{\eta }E_{\eta ,\eta +1}\left(-k_{n}t^{\eta }\right)\leq \frac{\Theta_{n}}{k_{n}}+\frac{\eta }{3}. \end{array}$$

Then, we can select appropriate design parameters such that (Θ_*n*_/*n*) ≤ (*η*/3). Therefore, from (74), (77), and (79), the following inequality can be obtained:

(79) $$\begin{array}{c} \left|V_{n}\left(t\right)\right|<\eta . \end{array}$$

According to (80) and the definition of *V*_*n*_(*t*), all signals in the closed-loop system are bounded and the tracking error *e*_1_(*t*) tends to an arbitrary small region of the origin, i.e., for all *t* > max{*t*_*r*_, *t*_*b*_}, such that (1/2)*e*_1_^2^(*t*) ≤ *η*.

## 4. Simulation Results

In this section, two examples will be given to demonstrate the effectiveness of the adaptive neural network backstepping control based on the command filtering.

**Example 1 .**

**Figure 1:**
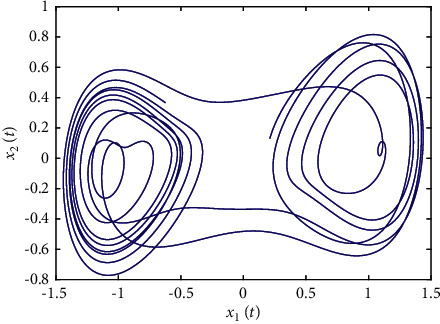
The uncontrolled fractional-order Duffing's oscillator chaos system (81).

**Figure 2:**
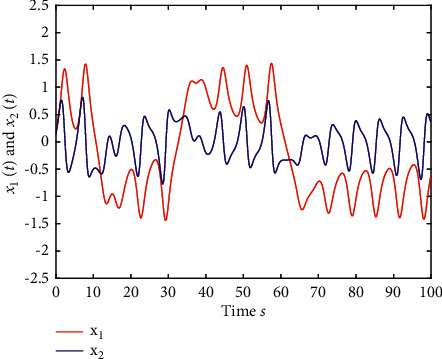
The time response of the state variable of (81) when *u*(*t*)=0 and *d*(*t*)=0.

The fractional-order Duffing's Oscillator chaos system is as follows:

(80) $$\begin{array}{c} \left\{\begin{array}{c} D_{t}^{\beta }x_{1}\left(t\right)=x_{2}\left(t\right),\\ D_{t}^{\beta }x_{2}\left(t\right)=x_{1}\left(t\right)-x_{1}^{3}\left(t\right)-0.5x_{2}\left(t\right)+1.3\;\cos t+d\left(t\right)+u\left(t\right), \end{array}\right) \end{array}$$

in which *f*_1_(*x*_1_(*t*))=0 and *f*_2_(*x*_1_(*t*), *x*_2_(*t*))=*x*_1_(*t*) − *x*_1_^3^(*t*) − 0.5*x*_2_(*t*)+1.3  cos*t* are unknown functions. Let the fractional-order *β*=0.95 and the initial conditions *x*_1_=0.21  and *x*_2_=0.13. In addition, it can be seen from Figures 1 and 2 that the nonlinear system is unstable when both the controller *u*(*t*) and the disturbance signal *d*(*t*) of the system are zero.

The known smooth reference signal *x*_*d*_(*t*) and the unknown external disturbance signal *d*(*t*) are chosen as sin(*t*) and sin(*t*)+cos(*t*), respectively. The parameters to be designed are selected as follows: *k*_11_=*k*_12_=1, *k*_21_=*k*_22_=1, *ρ*_1_=*ρ*_2_=5, *γ*_1_=*γ*_2_=0.5, *ξ*_1_=*ξ*_2_=1, and *κ*_1_=50. In the process of designing the controller, sign(·) is used to represent arctan(10) to avoid the chattering phenomenon.

Next, let us design the radial-basis-functions. According to the property of the radial-basis-function NN, the input variable is *x*_1_(*t*) in the first radial-basis-function and six Gaussian functions evenly distributed within the interval [−5,5] are designed. The second radial-basis-function uses *x*_1_(*t*) and *x*_2_(*t*) as input variables. Same as the first radial-basis-function, each input variable corresponds to six Gaussian functions evenly distributed on [−5,5]. The initial conditions are presented to be *θ*_1_(0)=[1,1,1,1,1,1]^*T*^ ∈ ℛ^6^ and *θ*_2_(0)=[1,1,…,1]^*T*^ ∈ ℛ^36^.

**Figure 3:**
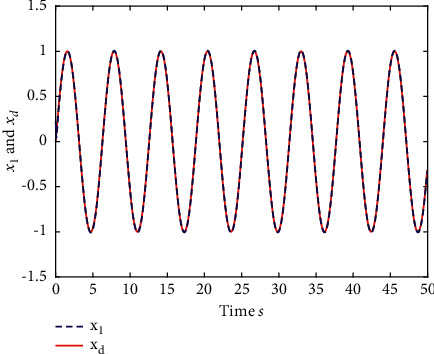
The trace of *x*_1_ and *x*_*d*_ of system (81).

**Figure 4:**
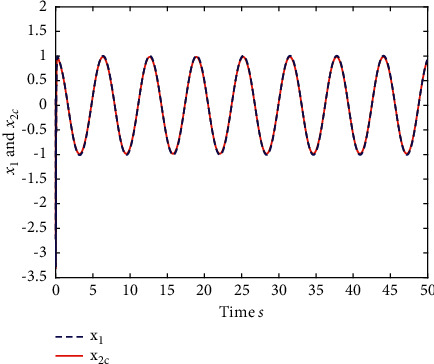
The trace of *x*_2_ and *x*_2*c*_ of system (81).

**Figure 5:**
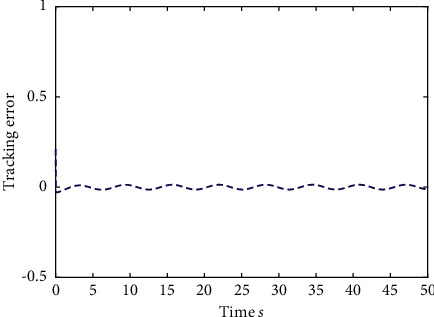
The tracking error of the output *y*(*t*) of system (81).

**Figure 6:**
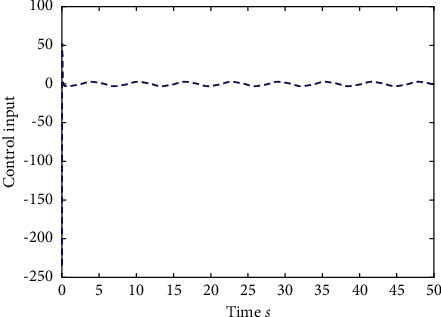
The control input of system (81).

**Figure 7:**
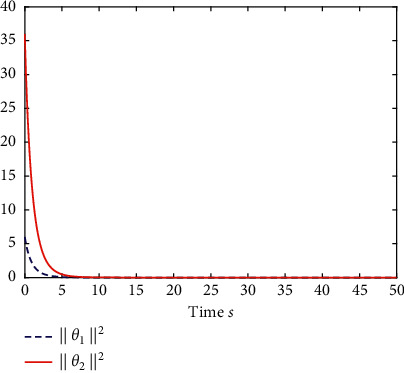
The adaptive laws of system (81).

When the above initial conditions are met, the simulation of the nonlinear system (81) is shown in Figures 3–7.

As can be seen from Figures 3 and 4, the state variables *x*_1_(*t*) and *x*_2_(*t*) of the system are tracked by signals *x*_*d*_(*t*) and *x*_2*c*_(*t*) and the tracking effect is relatively well. As shown in Figure 5, the tracking error *e*_1_(*t*) rapidly converges to a relatively small neighborhood of the origin. From Figure 6, the control input is large at first, then decreases rapidly, and when the system reaches stability, it is controlled in a relatively small neighborhood. As shown in Figure 7, the adaptive laws of the system are bounded and converge rapidly to the neighborhood of the origin. Consequently, the effectiveness of the adaptive neural network backstepping control method based on command filtering for a class of classical fractional-order nonlinear strictly feedback systems.

**Example 2 .**

**Figure 8:**
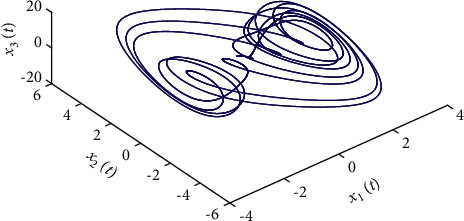
The uncontrolled fractional-order Arneodo's chaos system (81).

**Figure 9:**
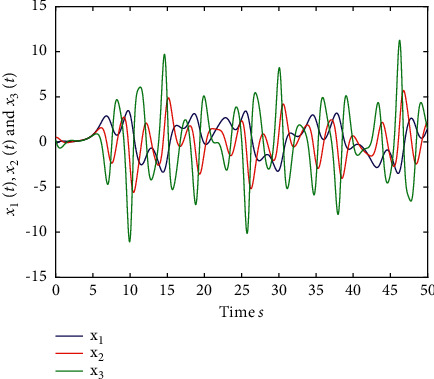
The time response of the state variable of (81) when *u*(*t*)=0 and *d*(*t*)=0.

The fractional-order Arneodo's chaos system is as follows:

(81) $$\begin{array}{c} \left\{\begin{array}{c} D_{t}^{\beta }x_{1}\left(t\right)=x_{2}\left(t\right),\\ D_{t}^{\beta }x_{2}\left(t\right)=x_{3}\left(t\right),\\ D_{t}^{\beta }x_{2}\left(t\right)=5.5x_{1}\left(t\right)-3.5x_{2}\left(t\right)-0.8x_{3}\left(t\right)+x_{1}^{3}\left(t\right)+d\left(t\right)+u\left(t\right), \end{array}\right) \end{array}$$

where *β*=0.97, *x*_1_(0)=−0.2,  *x*_2_(0)=0.5,  and *x*_3_(0)=0.2. If the input and disturbance of the system satisfy *u*(*t*)=0 and *d*(*t*)=0, the state variables of the system are shown in Figures 8 and 9.

The known smooth reference signal *x*_*d*_(*t*) and the unknown external disturbance signal *d*(*t*) are sin(*t*) and 2  sin(*t*)cos(*t*), respectively. The parameters to be designed are selected as follows:*k*_11_=40, *k*_12_=25, *k*_13_=30, *k*_21_=*k*_22_=*k*_23_=15, *ρ*_1_=*ρ*_2_=*ρ*_2_=15, *γ*_1_=*γ*_2_=*γ*_3_=0.5, *ξ*_1_=*ξ*_2_=1, *κ*_1_=1, and *κ*_2_=20.

**Figure 10:**
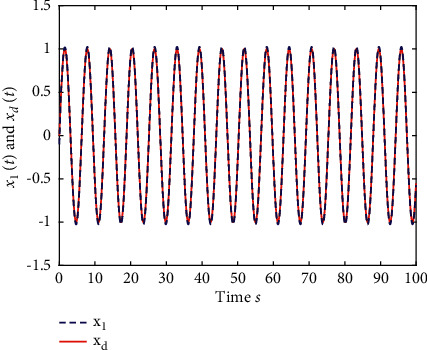
The trace of *x*_1_ and *x*_*d*_ of system (81).

**Figure 11:**
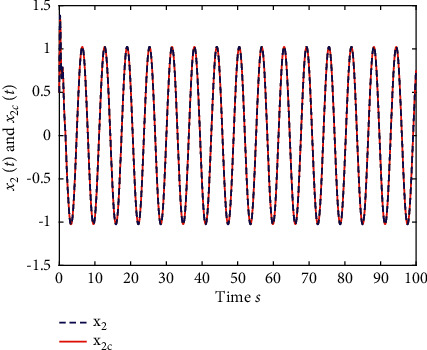
The trace of *x*_2_ and *x*_2*c*_ of system (81).

**Figure 12:**
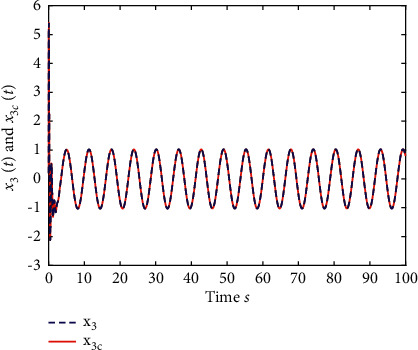
The trace of *x*_3_ and *x*_3*c*_ of system (81).

**Figure 13:**
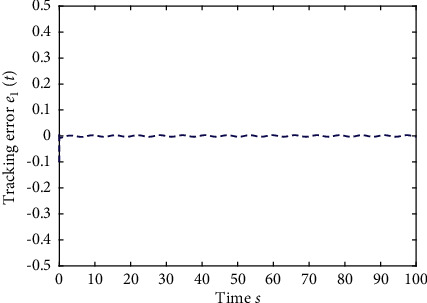
The tracking error of the output *y*(*t*) of system (81).

**Figure 14:**
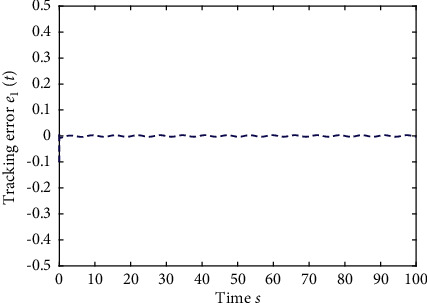
The control input of system (81).

**Figure 15:**
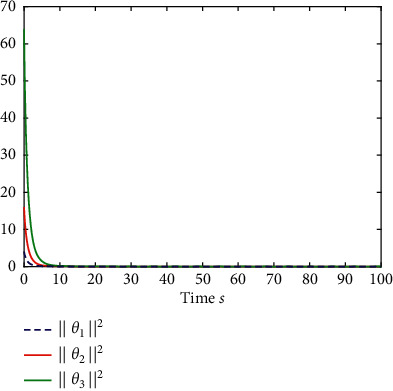
The adaptive laws of system (81).

In Example 2, there are three radial-basis-function neural networks. In the first radial-basis-function NN, the input variable is *x*_1_(*t*) and four Gaussian functions evenly distributed within the interval [−3,3] are designed. The second radial-basis-function uses *x*_1_(*t*) and *x*_2_(*t*) as input variables. Same as the first radial-basis-function, each input variable corresponds to four Gaussian functions evenly distributed on [−3,3]. The third radial-basis-function uses *x*_1_(*t*), *x*_2_(*t*), and *x*_3_(*t*) as input variables. Same as the before radial-basis-functions, four Gaussian functions in every input variable are evenly distributed on [−3,3]. The initial conditions are presented to be *θ*_1_(0)=[1,1,1,1]^*T*^ ∈ ℛ^4^, *θ*_2_(0)=[1,1,…,1]^*T*^ ∈ ℛ^16^, and *θ*_3_(0)=[1,1,…,1]^*T*^ ∈ ℛ^64^. When the above initial conditions are met, the simulation of the nonlinear system (81) is shown in Figures 10–15.

The simulation results of Example 2 are shown above, which is similar to the result analysis of Example 1, and the final results are the same as that of Example 1.

## 5. Conclusion

In this paper, an adaptive neural network backstepping control method based on command filtering is proposed for a class of fractional-order strictly feedback nonlinear systems. The command filtering technology is used to deal with the explosive of terms in the traditional backstepping technology, and the error compensation mechanism is introduced to overcome the shortcomings of the dynamic surface method. The simulation results of the fractional-order nonlinear strictly feedback system show the effectiveness of the proposed method. The future research will be adaptive neural network control of fractional-order nonlinear strictly feedback system with disturbance observer based on command filtering.

## Data Availability

All the datasets generated for this study are included within the article.
